# Potency of botulinum toxin drugs: test systems and labelling

**DOI:** 10.1007/s00702-022-02535-z

**Published:** 2022-08-30

**Authors:** Dirk Dressler, Eric A Johnson

**Affiliations:** 1 Movement Disorders Section, Department of Neurology, Hannover Medical School, Carl-Neuberg-Str. 1, 30625 Hannover, Germany; 2 Department of Bacteriology Botulinum Toxins Laboratory, University of Wisconsin Madison, WI, USA

**Keywords:** Botulinum toxin, Therapy potency testing, Potency labelling

## Abstract

Cell-based assays are a novel method to determine potency of botulinum toxin drugs. Manufacturers are working on their acquisition, development and implementation to reduce animal consumption during the manufacturing process. Potency labelling of botulinum toxin drugs differes principally between Ipsen and the other manufacturers. Reference to a uniform international standard would avoid this potentially dangerous situation. However, this has not been demanded by the registration authorities and has not been persued by the manufacturers for decades.

We thank the authors of this communication for conveying the views of Ipsen, one of the botulinum toxin manufacturers. They have sent different versions of their communication to the journal. We are responding the most recent one.

We are aware that Ipsen had tried to gain access to a cell-based assay through various partnerships and we have seen Ipsen’s poster presentation on BioSentinel’s cell-based assay. However, we have so far no independent confirmation, that such a cell-based assay has actually replaced their mouse lethality assay. The quoted press release announces a mere intention to do so and the quoted patient web site mentions a cell-based assay only as a ‘primary release procedure’. If Eskenazi et al. should have a mandate to speak for Ipsen, we and the readers of this journal would be glad to note, that the company has abolished animal testing for their drug manufacturing.

We have always explicitly stated (and we have done so in the current article), that the potency labelling of different botulinum toxin drugs is not identical. We would be grateful, if the authors of this communication would acknowledge this. We have asked all botulinum toxin manufacturers for decades to refer their potency labelling to one international standard. To much of our surprise, all of them have refused to do so. It may be speculated, that this was done to generate myths about ‘uniqueness’ of products and to inhibit fair comparisons of efficacy, safety and prices. To provide these crucial data, the international botulinum toxin user community has developed conversion ratios for most botulinum toxin drugs. Only for abobotulinumtoxinA, these conversion factors are still a matter of debate. This leaves this drug and its potency labelling (and possibly Masport^®^, an Iranian abobotulinumtoxinA clone) isolated from the rest of the botulinum toxin world.

Besides, we would be much interested to learn, why potency units would ‘become obsolete with recombinant technologies’.

The community would certainly very much welcome, if Ipsen would make its points in a peer-reviewed publication.

